# Future Information Technology Tools for Fighting Substandard and Falsified Medicines in Low- and Middle-Income Countries

**DOI:** 10.3389/fphar.2018.00995

**Published:** 2018-08-31

**Authors:** Huma Rasheed, Ludwig Höllein, Ulrike Holzgrabe

**Affiliations:** ^1^Institute of Pharmacy and Food Chemistry, University of Wuerzburg, Wuerzburg, Germany; ^2^Institute of Pharmaceutical Sciences, University of Veterinary and Animal Sciences, Lahore, Pakistan

**Keywords:** information technology, substandard and falsified medicines, field testing, quality evaluation, mobile apps, medicine authentication tools, Track and Trace

## Abstract

Substandard and falsified (SF) medicines have emerged as a global public health issue within the last two decades especially in low- and middle-income countries (LMICs). Serious consequences of this problem include a loss of trust and increased financial costs due to less disease control and more frequent complications during therapy. Of note, antimicrobial resistance is an additional long-term implication of poor-quality antimicrobials. This review covers information technology tools including medicines authentication tools (MAT) as mobile apps and messaging service, 2D barcoding approaches with drug safety alert systems, web based drug safety alerts, radiofrequency identification tags, databases to support visual inspection, digital aids to enhance the performance of quality evaluation kits, reference libraries for identification of falsified and substandard medicines, and quality evaluation kits based on machine learning for field testing. While being easy to access and simple to use, these initiatives are gaining acceptance in LMICs. Implementing 2D barcoding based on end-to-end verification and “Track and Trace” systems has emerged as a step toward global security in the supply chain. A breakthrough in web-based drug safety alert systems and data bases was the establishment of the Global Surveillance and Monitoring System by the World Health Organization in 2013. Future applications include concepts including “lab on a chip” and “paper analytical devices” and are claimed to be convenient and simple to use as well as affordable. The principles discussed herein are making profound impact in the fight against substandard and falsified medicines, offering cheap and accessible solutions.

## Introduction

Low- and middle-income countries (LMICs) are frequently affected by the pandemic of substandard and falsified (SF) medicines. Three quarters of the samples included in the latest World Health Organization (WHO) review were collected in LMICs and show a percentage failure rate of 9.9–10.9% amounting to a crude worth estimate of 30.5 billion USD per annum (World Health Organization, 2017a). In May 2017, the WHO revised the definition of SF medical products, whereby the term “counterfeit” has been withdrawn from this context (World Health Organization, 2017b). In brief, SF medicines are differentiated, the latter referring to unclear identity, composition, or source of the respective product.

Among the various solutions and strategies devised for combating poor-quality medicines (Kovacs et al., 2014; Höllein et al., 2016), a substantial portion is based upon information technology tools for fighting falsified medicines (Mackey and Nayyar, 2017). Information technology is defined by Merriam-Webster dictionary as *“the technology involving the development, maintenance, and use of computer systems, software and networks for the processing and distribution of data “* (Merriam Webster Dictionary, 2018). A variety of technologies and approaches has been published for the detection of falsified and substandard medicines in LMIC and resource limited settings (Kaur et al., 2010; Glass, 2014; Kovacs et al., 2014; Höllein et al., 2016) which have also been compared with regard to costs, simplicity, and performance (Glass, 2014; Kovacs et al., 2014; Batson et al., 2016). In general, they are easy to operate and access, exhibit low costs, and are very user-friendly (Kovacs et al., 2014). The methods are used for initial identification, quality evaluation, as well as an efficient dissemination of information on SF medicines. The WHO proposed a three-pronged approach of prevention, detection, and response in order to tackle the menace of SF medical products (World Health Organization, 2017c). This review substantiates the above mentioned WHO approach and covers information technology tools including Medicines Authentication Tools (MAT) as mobile apps and messaging service, 2D barcoding linked MAT with drug safety alert systems, web based drug safety alerts, Radio Frequency Identification Tagging (RFID) for product tracking, databases for identification of medicinal products to aid visual inspection, digital aids to enhance performance of quality evaluation kits, reference libraries for identification of falsified and substandard medicines employing various analytical techniques, as well as quality evaluation kits based on machine learning for cheap and convenient field testing.

## Classification of it Tools for SF Medical Products based on USAGE

All identified information technology tools were classified into five categories based on their field of application (**Table 1**). The type of tool (mobile application, database, software, or field testing kit), respective examples, its role, and the relevance for the end user are incorporated against each application category (**Table 1**).

**Table 1:** Information technology tools used for the identification and control of falsified and substandard medicines and medicinal products.

Category/Application	Name	Function	End user
**Supply chain tracking systems**		
Mobile apps	*mPedigree 2006*	Product authenticity	Up to patient
	*Sproxil 2009*	Product authenticity	Up to patient
	*Pharmasecure 2007*	Product authenticity + reporting of suspect SF medicine	Up to patient, regulator
	*Epothecary*	Product authenticity	Up to patient
	*Medsnap (2011)*	Product authenticity	Up to patient
Software + igital repository + Mobile App (based on block chain technology)	Aegate Holdings Limited	2D barcoding and end-to- end verification	Manufacturer and each change of ownership in supply chain up to patient.
	Arvato Systems GmbH		
	Solidsoft Reply		
Radio frequency readable tagging of product packs	*RFID*	Product authentication and tracking to unauthorized markets	Manufacturer/regulator
Edible Radio frequency readable tagging of unit doses	*TruTag^®^*		
**Information dissemination**		
Database	*Global Surveillance for Monitoring System (GSMS)*	WHO global data base for reporting and processing of information on SF medicines	Global regulators-open access
Database	*Medication Quality Database (MDQB)*	USP data base for reporting and processing of information on SF medicines	Global regulators-open access
**Quality evaluation**		
Mobile app		To improve visual assessment of spots in MiniLab^®^ TLC kits	Pharmacy mangers and regulators in field testing
Lab on a Chip	Pharmacheck	Field testing kit	
Paper analytical devices	Paper analytical devices	Field testing kit	
Spectral libraries	Electronic database for reference spectra and fingerprints with or without machine learning	For identification of falsified medicines using Raman, IR, NIR and CD3+ in lab or field set up.	
Digital libraries for physical inspection	Identa (https://www.gelbe-liste.de/identa)	Field testing, regular physical audits	

### Supply Chain Tracking Systems

These are mainly information technology-based solutions used for examining the intact packages at various points within the supply chain up to the consumer level. The concept of end-to-end verification and “Track and Trace” is characteristic for all these services, forming the fundamental basis of a traceable supply chain surveillance. Such systems gain more and more global attention.

#### Medicines Serialization and Verification Technology

Medicines are coded using a unique 12-digit serialization and a 2D barcode system with an end-to-end “Track and Trace” process comprising of the following steps (Naughton et al., 2017):

(a) Systematic serialization of the products at the manufacturing site. The serialization code uses block chain technology to enable end-to-end decryption (manufacturer to each user in supply chain) offering retrieval of information by an ePedigree-based digital ledger (Mackey and Nayyar, 2017). The individual coding applies to both raw materials and finished pharmaceutical products and allows multiple participants to update tracking information, to authenticate, and also to share information using integrated anticounterfeit devices (Mackey and Nayyar, 2017).
(b) Authentication, i.e., verification of the product authenticity, by scanning the imprinted codes reveals the “authenticity status” and may include additional information, e.g., whether the product is expired, recalled or falsified. The *European Union Falsified Medicines Directive* (EU-FMD) requires this authentication to be carried out at the point of supply, whereas the *United States Drug Supply Chain Security Act* (DSCSA) demands a verification on each change of ownership during the medicine supply and distribution process, respectively.
(c) Decommissioning of the product, if applicable, is required at the final point of supply to the patient in accordance with EU-FMD.

As soon as a product has been scanned for verification, a color-coded pop-up message appears, guiding the user to take appropriate action, e.g., whether a product must be quarantined or can be handed to the patient, respectively. Such warnings and alerts may also be implemented during stocking, dispensing, and supply of medicines, facilitating the identification of suspected or faulty stocks.

The effectiveness of the system is based upon the practice of the end users, i.e., all medicines requiring authentication must be authenticated eventually and a respective action must be taken, accordingly (Naughton et al., 2017). Examples for technology providers are Aegate Holdings Limited, Arvato Systems GmbH, and Solidsoft Reply; they have been assigned by *The European Medicines Verification Organization* for the provision of medication authentication technology within the European Union (Naughton et al., 2017).

“Track and Trace” systems using 2D or data matrix bar coding are gaining world-wide acceptance. **Table 2** summarizes the implementation status in different countries. In January 2012, Turkey became the very first country to implement a fully mobile app based verification system (Taylor, 2015). SecurPharm, a consortium of the *Federal Union of German Association of Pharmacists* (ABDA) and other professional organizations, launched its pilot project in 2013 involving pharmaceutical manufacturers, wholesalers, and about 400 pharmacies (securPharm e.V, 2012, 2016, 2018). The EU-FMD marks 9th February 2019 as deadline for adopting this technology on all prescription medicines throughout all member states (European Medicines Agency, 2017).

**Table 2:** Implementation status for 2D bar coding of medicines in different countries.

Region/Country	Directive/Legislation/Authority	Issue date	Implementation deadline for 2D barcoding
European Union and United Kingdom	EU Falsified Medicines Directive (EU-FMD) (European Medicines Agency, 2011)	2011	
European Union	Commission Delegated Regulation (EU) 2016/161 (The European Commission, 2016)	9th February, 2016	9th February, 2019
United States	US Drug Supply Chain Security Act (DSCSA) (US Food and Drug Administration, 2017a)	27th November, 2013	Serialization: November, 2018 (revised) complete enforcement: late 2023
China	Serialization Mandate (CFDA) (China Food and Drug Administration, 2015)	21st October, 2015 enactment date 1st February, 2016	
Brazil	National Agency for Sanitary Surveillance in Brazil (ANVISA) (Infosys Limited, 2017)	2009 serialization initiation 2013 draft legislation 2014 final proposal	
New Zealand	NZ Health information standards (GS1, 2012)	August 2011	
Canada	Joint Technical Statement on Canadian Pharmaceutical Automated Identification and Product Data Requirements	January 2010, revised February 2012	1st December, 2017 (ISMP Canada, 2013) For vaccines (Government of Canada, 2016) Primary level: January 2017 Secondary pack: January 2018
India	Drug authentication and verification application (DAVA) (Trace-Links Life Sciences Cloud, 2018)	July 2015	1st October, 2015 revised to March 2016 and 2017
Pakistan	Amendments in drug (Labeling and Packaging rules), 1986 (Part I-III) system (Finance Division Government of Pakistan, 2017)	June, 2015	June, 2019
Turkey, Argentina, France, South Korea	Already achieved		

#### Mobile Product Authentication

Mobile applications for medication authentication are accessible for large parts of the population, thus directly reaching the patient. Mobile apps and SMS based medicine authentication tools for detecting falsified medicines were pioneered by mPedigree (Nigeria) in 2006 (Wall, 2017), Sproxil (Nigeria) in 2009 (Wall, 2017), İlaç Takip Sistemi (Turkey) in 2010, and Pharmsecure (Nigeria and India) in 2012. These MATs involve the use of a visible or scratchable code which has been printed on the product package by the manufacturer. The patient sends this code to the respective authentication databases using SMS. In reply, a message is received stating whether the stocks inquired are tagged genuine or fake; scannable bar codes are also provided. In Pharmsecure, customers can also capture a photograph of the suspected medicine and send it to the database administrators. Evidence is subsequently transferred to the manufacturer for further investigation. Hence, an early detection of SF medicines is possible, facilitating a rapid response.

The mobile App MyDawa from Ion Kenya, Inc. also uses scratchable codes and can be used for verifying the authenticity of medicines and related medicinal supplies delivered to the Nigerian market. More than 70 international pharmaceutical companies are using the services of Sproxil in Kenya, Ghana, Nigeria, South Africa, and Mali (Wall, 2017). The mPedigree startup was initiated in Nigeria and is now operational in 12 countries across Asia and Africa.

Physicochemical identifiers are a form of authentication token using invisible imprints on a distinct product unit (Wall, 2017). For example, the TruTag^®^ technology employs edible micro-tagging of tablets using high purity silica which offers low costs and reliable solutions for improved security measures for products and/or medicines prone to falsification and illegal trafficking (TruTag Technologies Inc., 2017). The product information can be retrieved anywhere using portable TruTag^®^ optical scanners (TruTag Technologies Inc., 2017).

#### Radio Frequency Identification (RFID)

RFID tags are applicable to products and respective packages using active or passive chips being able to deliver a small set of information, e.g., regarding the origin of a particular product (ISMP Canada, 2013). Usually, such systems consist of a transponder or a tag affixed to or carried in the product, transmitting various information to the interrogator or the reader in form of radio waves or wireless signals, respectively. All fields of logistics can benefit from this technique; commonly, passive tags are used which transmit data only when irradiated with a radio signal from an RFID reader in close proximity, thus not requiring any power supply. Modern hardware is designed in a very inconspicuous manner and is mostly constructed as thin labels or small microchips which can be placed inside a product packaging, thus being invisible at first glance but readable from outside.

Although using this technology is recommended by the *U.S. Food and Drug Administration*, the individual costs per unit are quite high when compared to optical coding techniques. However, particularly in the field of monitoring temperature sensitive medicines, active chips are utilized to constantly record the surrounding temperature and thus, to provide a detailed log at any time during and after transport as well as storage.

### Databases for Information Processing and Dissemination

The use of drug safety databases and alerts regarding SF medicines has become an essential tool for an efficient and reliable dissemination of information and control of poor-quality medicines in the global medicine supply chains. A remarkable breakthrough in this context is the establishment of the *Global Surveillance and Monitoring System* (GSMS) by the WHO in 2013. Recent landmark reports on SF medicinal products conducted by the WHO presented a literature review of 100 publications with major data contributed from the *Medicines Quality Database* (MQDB) and the GSMS (World Health Organization, 2017a).

### Quality Evaluation in Field Testing (on Spot and In-Time Results)

Routine quality evaluation procedures require a sophisticated and expensive infrastructure which prove to be a limitation for LMICs (Höllein et al., 2016). A few novel approaches of developing mobile, convenient, and affordable field kits using machine learning to identify and quantify a medicine based upon its chemical nature (Mackey and Nayyar, 2017) or a unique finger print derived from drug interaction analysis are under development (Redaktion Gelbe Liste, 2013; Weinstein and Zaman, 2017).

#### Improving Performance of GPHF MiniLab^®^ Through Mobile Application

The GPHF MiniLab^®^ tool kit, established in the 1980’s by the Global Pharma Health Fund, pioneers the concept of field testing for detection of counterfeit and substandard medicines. It has been operating in 95 countries across the world. The current edition of the MiniLab^®^ manual covers more than 90 active compounds including essential antibacterial and antituberculotic drugs. The kit employs thin layer chromatography with visual evaluation of the respective chromatograms as main analytical technique. Of note, the tests are semiquantitative and thus not suitable for analyzing the content of compounds where the exact dosage is critical, e.g., in the case of antibiotics. However, the limitation of visual evaluation has recently been overcome by introducing a mobile phone application for measuring and comparing the spot intensity (Fadeyi et al., 2015). In addition, it was shown that during analysis and evaluation, significant deviations from the true content might occur (World Health Organization, 2011; Höllein and Holzgrabe, 2014).

#### Pharmacheck

The project aims to deliver a “lab on a chip” concept that can give results by color indicators in the field (Beltman et al., 2013; Weinstein and Zaman, 2017). Interaction analysis was performed using *E. coli* to develop fingerprint results for 27 antibiotics. The project is in its developmental stage and aims to provide a fast, low cost, and portable technology that is applicable to the most remote settings. No sample preparation, electric supply, and only minimal training is required (Kovacs et al., 2014).

#### Use of Spectral Libraries for Vibrational Spectroscopy

Spectroscopic libraries are required in Raman and near infra-red (NIR) spectroscopy as well as in newer technologies like the CD^3+^ counterfeit device approved by the U.S. Food and Drug Administration or Paper Analytical Devices. These techniques may or may not be linked to machine learning tools for the identification of SF medicines (Kovacs et al., 2014; Batson et al., 2016; Mackey and Nayyar, 2017), exhibiting low to medium costs and representing valuable tools for detecting SF medicines (Kovacs et al., 2014; Batson et al., 2016; Mackey and Nayyar, 2017).

#### Use of Digital Libraries for Aiding Visual Inspection

Visual inspection of the primary and secondary product packages as well as the respective dosage forms holds a key position in immediate identification of falsified medicines. A respective scheme is offered within a WHO guideline (World Health Organization, 1999; Kaur et al., 2010). In Germany, registered pharmacists and practitioners have access to the online software identa^1^ for physical identification of medicaments, thus facilitating an early identification and reporting of suspicious medicines. The recall of falsified Pegasys^®^ injection in Germany in 2013, identified during the routine internal audit process, is a prominent example of unraveling falsified medicines using this tool (Redaktion Gelbe Liste, 2013; Roche Pharma AG, 2017).

## Discussion and Conclusion

The constant rise of various types of falsified and substandard medicines demand the incorporation of a fast and effective identification of poor-quality medicines throughout the global supply chain. Efficient and reliable processing of information as well as dissemination of alerts is warranted to minimize the damage caused by the administration of any faulty medicines. Information technology has placed a significant role in providing solutions to both these needs. The majority of currently available applications comprise of medicine authentication tools involving verification of the product packaging. Identification of falsified medicines based upon their active pharmaceutical ingredient (API) content is also anticipated. However, very few applications claim to inform on the amount and purity of the API and innovative approaches are needed to offer accessible solutions in this regard.

Implementing 2D and data matrix barcoding has come up as a unified global strategy. Countries have placed this global intervention program within their respective health system at varying pace. Phase wise realization is seen, with the first step being verifying the serialization and data banking at a local repository (fixed data), proceeding to the second level of enabling data access using a cloud-based repository accessible by multiple users to receive, share, and update information. Equipping pharmacies with the respective soft- and hardware devices including barcode scanners is also a major part of this process. The final stage of the “Track and Trace” process enables a proactive bidirectional system of sending alerts and product warnings that could be linked to electronic prescribing portals to ensure maximum patient safety. However, costs and lack of awareness about the barcoding system had been the major impeding factors in its implementation process. The United States have postponed (US Food and Drug Administration, 2017b) the process to several deadlines and so is the case with Pakistan, where a large manufacturing industry exists. It is yet to achieve the first milestone of elevating a *Global Trade Identification Number* (GTIN) and serialization to the level of primary packaging by all manufacturers.

India and China are the two main global suppliers of raw materials and finished pharmaceuticals, and their compliance with the implementation of GS1 standards using barcoding holds crucial importance in the success of an end-to-end “Track and Trace” process. The Indian *Drug Authentication and Verification Application* (DAVA) introduced in 2012 was an award-winning project (GS1 India, 2017). Contrary to that, China has not yet adopted the global concept of serialization and a separate *China National Drug Code* with serial numbers is issued through its own *Product Identification, Authentication and Tracking System* (PIATS) (Infosys Limited, 2017).

Implementation of product serialization and barcoding enables end users, including patients, to identify the authenticity of a product, e.g., through mobile phones equipped with a suitable application for reading the barcodes. This broader accessibility of product identification tools down to the patient level will create more public awareness and a participatory approach for the identification of poor-quality medicines in the supply chain. Increased sensitivity at the consumer level and their engagement in implementing a collective scrutiny solution regarding falsified medicines (Mackey and Nayyar, 2017) is particularly important for LMICs where most of the health expenditure is covered by out-of-pocket expense.

“Track and Trace” is a proactive system that updates consumer on the product safety and authenticity at each stage of usage. The collaboration of several systems (e.g., GSMS, Vigibase, and “Track and Trace”) will provide a major impact in the delivery of safe and efficacious medicines worldwide. GSMS and Vigibase are two major global data bases, with GSMS targeting only registration of SF medicines, whereas Vigibase is based upon pharmacovigilance. Currently, LMICs are only minor contributors to the global data base on pharmacovigilance. The implementation of GSMS in the WHO member states will increase the sensitization of authorities and masses over drug safety issues.

Certain regions and countries face poor access to medicines due to failure of registration status, unavailability of particular products, high potentials for drug abuse, corruption or pricing issues, all of them representing predisposing factors to illegal medicine trade and diversion (World Health Organization, 2017a). Tracking is inevitable to ensure that funded and/or donated supplies reach their intended destinations, of note particularly including conflict and disaster-ridden regions. RFID is the best resource for tracking any unauthorized movement of medicines because of the unique technology design and the possibility to extract and store the respective information in web-based portals or server systems at any time (Mackey and Nayyar, 2017). In contrast to 2D barcoding, RFID technologies can be used to identify a product even if it is not in the line-of-sight of the scanner and is detected automatically when in range of the receiver (ISMP Canada, 2013). Readers can be portable, mounted on a post or over head or built in the construction of storage space or buildings, (US Food and Drug Administration, 2017c) thereby increasing the chances of detection of unauthorized movement of the product or consignment. Moreover, radiowaves can penetrate several layers of packaging and allow batch reading of multiple items at a time (ISMP Canada, 2013).

The availability of digital product identification catalogs like “identa” for making regular internal audits for stocked medicines invites retail pharmacist to share the role of a regulator in identification of falsified medicines (Gelbe Liste, 2018). This cost-effective, proactive, and participatory model of fighting against SF medicines practiced in Germany can prove to be effective in LMICs. Availability of such software to health care professionals through mobile applications can make it more user friendly and applicable in remote settings. Training of the field regulatory inspectors on use of such aids can enhance the impact of the intervention. Information technology and digital aids are having a profound impact in the fight against SF medicines and constitute a promising area offering cheap and broadly accessible solutions.

## Author Contributions

HR conceived, designed the review, and wrote first draft of the manuscript. LH and UH refined the draft, and provided intellectual input. All authors contributed to manuscript revision, read, and approved the submitted version.

## Conflict of Interest Statement

The authors declare that the research was conducted in the absence of any commercial or financial relationships that could be construed as a potential conflict of interest.

## Footnotes

1 https://www.gelbe-liste.de/identa
